# An Unusual Manifestation of HSV-1 Uveitis Transforming into an Acute Iris Transillumination-like Syndrome with Pigmentary Glaucoma: A Reminder of Treatment Pitfalls in Herpetic Uveitis

**DOI:** 10.3390/life15081164

**Published:** 2025-07-23

**Authors:** Marin Radmilović, Goran Marić, Ante Vukojević, Mia Zorić Geber, Zoran Vatavuk

**Affiliations:** Department of Ophthalmology, Sestre milosrdnice University Hospital Center, Vinogradska cesta 29, 10000 Zagreb, Croatia; radmilovic.marin@gmail.com (M.R.); goran.maric10@gmail.com (G.M.); ante.vukojevic1@gmail.com (A.V.); zo.vatavuk@gmail.com (Z.V.)

**Keywords:** Herpes simplex virus (HSV-1), anterior uveitis, acute iris transillumination, pigmentary glaucoma, antiviral therapy

## Abstract

We report a case of herpes simplex virus type 1 (HSV-1) anterior uveitis evolving into an acute iris transillumination-like syndrome with secondary pigmentary glaucoma, highlighting diagnostic challenges and treatment considerations. A 61-year-old immunocompetent woman presented with unilateral anterior uveitis characterized by keratic precipitates and mild anterior chamber inflammation. The condition was initially treated with topical and subconjunctival corticosteroids without antiviral therapy. After an initial resolution of symptoms, upon the cessation of treatment, the patient developed features resembling unilateral acute iris transillumination (UAIT) syndrome with elevated intraocular pressure, diffuse pigment dispersion, and progressive iris transillumination defects. Aqueous polymerase chain reaction (PCR) testing confirmed the presence of HSV-1. Despite the initiation of antiviral therapy, the condition progressed to severe pigmentary glaucoma, with unreliable intraocular pressure measurements due to prior LASIK surgery. Cataract extraction, pars plana vitrectomy, and Ahmed valve implantation were performed, with only partial recovery of visual acuity. This case illustrates that HSV-1 uveitis can mimic or transition into a UAIT-like syndrome, possibly due to steroid use without concurrent antiviral treatment, which may exacerbate viral replication and damage to the iris pigment epithelium. Aqueous PCR testing aids in differential diagnosis, but indicative medical history and clinical findings should remain instrumental. Clinicians should maintain a high index of suspicion for herpetic etiology in anterior uveitis cases and initiate prompt antiviral treatment to prevent potentially sight-threatening complications.

## 1. Introduction

Herpes simplex virus (HSV) has been established as a cause of acute anterior uveitis, usually with unilateral presentation, with possible sectoral iris atrophy, pigment dispersion, and secondary ocular hypertension [1,2,3]. The main differential diagnoses in such cases include pigment dispersion syndrome, Fuchs heterochromic iridocyclitis, and acute bilateral or unilateral iris transillumination syndrome (BAIT/UAIT). These entities can usually be distinguished with a careful evaluation of medical history, laterality, iris atrophy patterns, as well as the presence or lack of inflammatory findings, which are especially stressed when discerning herpetic uveitis from BAIT/UAIT [4]. The latter presents similarly to herpetic uveitis, albeit almost always bilaterally, with acute onset of ocular pain, redness, and photophobia. However, it is characterized by diffuse pigment dispersion from the iris pigment epithelium (IPE), its deposition in anterior chamber structures, and the absence of keratic precipitates, anterior chamber inflammatory cells, or vitritis [4,5,6]. While the etiopathogenesis of BAIT/UAIT is still not fully understood, it has been attributed to the toxic effect of systemic or local antibiotic therapy, most commonly fluoroquinolones, on IPE [7,8,9,10,11]. Given the existence of antecedent viral infections in two-thirds of these patients, viral etiology has also been suggested to play a role [4,12].

We report a case of acute anterior uveitis due to herpes simplex virus type 1 (HSV-1), developing clinically from a typical herpetic uveitis into an acute iris transillumination-like syndrome with pigmentary glaucoma, necessitating cataract surgery, vitrectomy, and glaucoma filtration surgery.

## 2. Case Report

A 61-year-old woman presented to the emergency department with redness, pain, and blurred vision in her left eye. Her ocular history included amblyopia in the right eye, and LASIK performed for high myopia 12 years prior on the left eye. Her systemic history included Hashimoto thyroiditis, an episode of pericarditis 4 years prior, and an episode of viral pneumonia 2 years prior. She was otherwise healthy, took no antibiotics or other medications, had no recent vaccinations, and experienced no viral prodrome or other symptoms. At the time of the initial evaluation, her best corrected visual acuity (BCVA) was hand motion (HM) in the right, amblyopic eye, and 20/40 in the left eye. Her intraocular pressure (IOP) measured 12 mmHg in the right eye and 14 mmHg in the left eye on Goldmann applanation tonometry (GAT). Her left eye was documented (only textually, with no available photodocumentation) to show ciliary injection, fine keratic precipitates, and anterior chamber cells, with normal pupils and no other pathological findings except for a mild nuclear cataract. No signs of vitritis were noted, and a dilated fundus examination showed a slightly tilted optic disc with myopic fundus tessellation and an inferior type of posterior staphyloma (Curtin type V) but was otherwise unremarkable. The right eye showed no signs of inflammation or any other pathological finding aside from a dense nuclear cataract, a slightly tilted optic disc, fundus tessellation, and a posterior pole staphyloma (Curtin type I).

**Figure 1:**
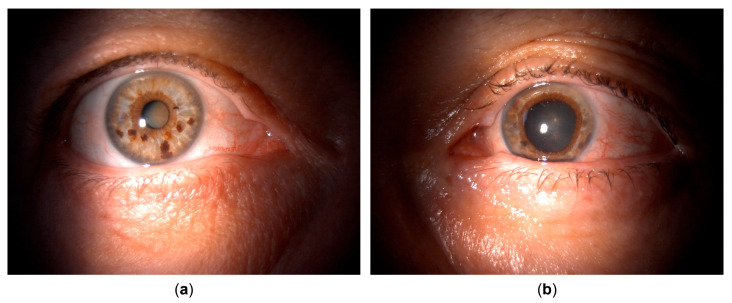
Clinical appearance upon referral to the uveitis service. (**a**) Unaffected (right) eye. (**b**) Affected (left) eye with irregular, mid-dilated, unresponsive pupil, mild bulbar hyperemia, pigmented corneal precipitates, and pigment deposits posterior to the crystalline lens.

**Figure 2:**
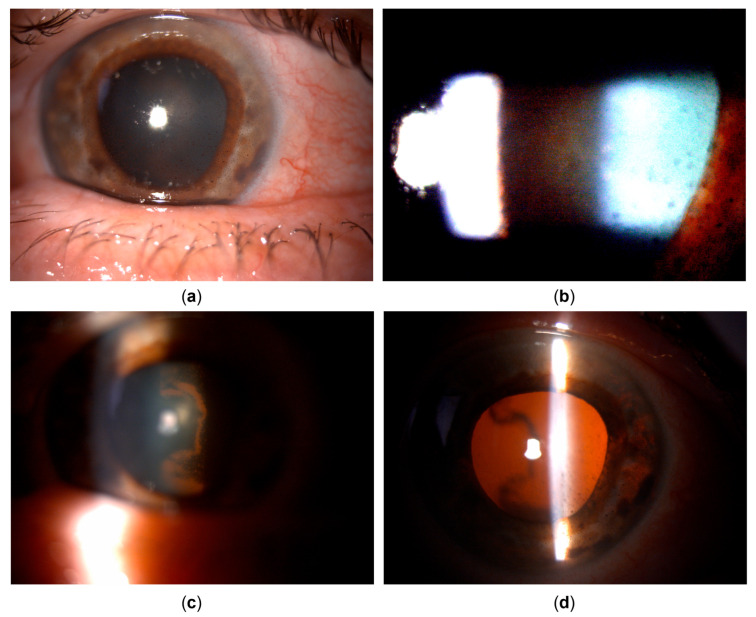
Clinical appearance upon referral to the uveitis service (details, left eye). (**a**) Pigmented corneal precipitates with fine and stellate morphology. (**b**) Marked pigment dispersion in the anterior chamber. (**c**) Pigment deposits on the anterior hyaloid surface and posterior lens capsule, indicating the temporal origin of pigment dispersion. (**d**) Iris transillumination defects showing sectoral, patchy morphology in the temporal portion of the iris.

The general ophthalmologist who saw the patient applied a subconjunctival dexamethasone injection and prescribed treatment with dexamethasone drops and ointment, tropicamide drops, and oral ibuprofen. Over the course of one month, the patient reported an almost complete resolution of symptoms; her left eye BCVA improved to 20/25, and signs of inflammation regressed, with only a few keratic precipitates and no cells observed in the anterior chamber. The treatment was tapered and subsequently discontinued. However, within a couple of days after treatment cessation, the patient returned complaining of blurred vision once again. Her left eye BCVA was now 20/50, and slit lamp examination revealed diffuse pigmented precipitates on the corneal endothelium and the anterior iris surface, an abundant convection current of dispersed pigment in the anterior chamber, and dense pigment deposition on the anterior hyaloid membrane and posterior lens capsule in the visual axis and temporally (Figure 1 and Figure 2). Additionally, the IOP of the left eye was now elevated and measured 31 mmHg. No corneal infiltration or non-pigmented keratic precipitates, anterior chamber cells, vitritis, or posterior synechiae were noted. The patient was administered another subconjunctival dexamethasone injection, restarted on topical dexamethasone and tropicamide, with added dorzolamide/timolol drops, and referred to a uveitis specialist.

Upon referral, a mid-dilated, irregular pupil with poor light reaction was additionally noted, with the iris showing pronounced sectoral transillumination defects in the temporal quadrant (Figure 1 and Figure 2). A working diagnosis of UAIT syndrome seemed possible for the abundance of pigment dispersion and a current lack of inflammatory cells in the anterior chamber or vitritis. However, given the likely differential diagnosis of herpetic uveitis, especially due to previously described findings and a good response to dexamethasone treatment, as well as a typical finding of sectoral iris transillumination and the stellate morphology of pigmented corneal precipitates, the patient was started on oral acyclovir (800 mg five times daily, which was planned to be maintained long-term) with continued topical therapy and dexamethasone injections. An anterior chamber tap for polymerase chain reaction (PCR) was performed to test for Herpes simplex virus type 1 and 2, the Varicella-Zoster virus (VZV), Cytomegalovirus (CMV), and Epstein–Barr virus (EBV), returning positive for HSV-1.

**Figure 3:**
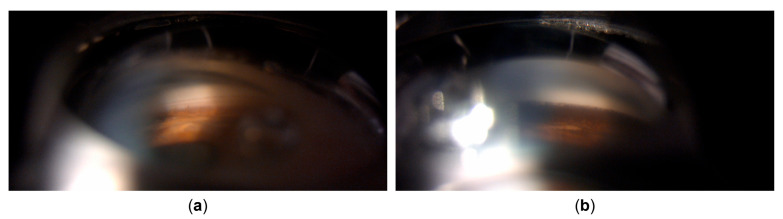
Gonioscopy. (**a**) Right eye showing open angle with mild pigmentation. (**b**) Left eye with heavy pigmentation obscuring all angle structures.

Despite the ongoing treatment, and despite the continuous lack of inflammatory cells in the anterior chamber or vitreous, BCVA further deteriorated to 20/400 with increasing retrolental pigment deposition. The transillumination defects progressed to encompass the entire iris, and gonioscopy showed an open angle with circumferential dense pigmentation obscuring all structures (Figure 3). Intraocular pressure mounted, prompting the addition of brimonidine and bimatoprost drops with oral acetazolamide, which maintained IOP variably between 14 and 28 mmHg, as measured by GAT. However, discrete retinal arterial pulsations were occasionally observed at the optic disc in the left eye, suggesting that the true IOP may have been higher than the measured values, especially considering the known challenges of accurate IOP measurement in post-LASIK eyes [13,14].

**Figure 4:**
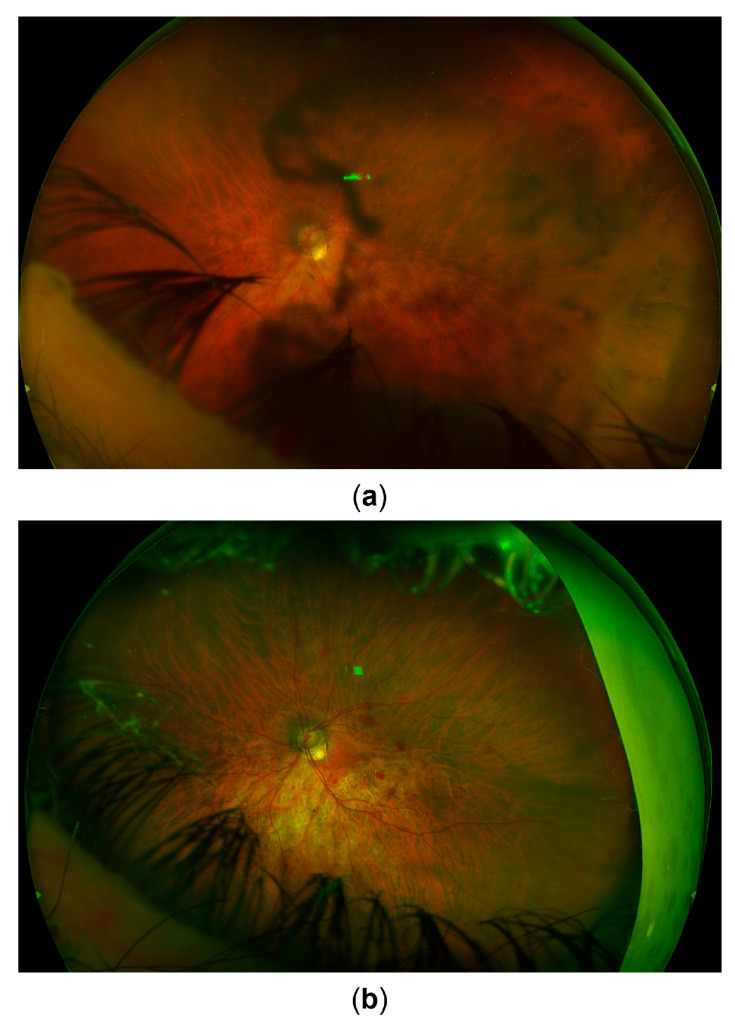
Fundus image. (**a**) Appearance upon referral to the uveitis service. (**b**) Appearance 3 months later after recovery from combined cataract surgery and vitrectomy. Signs of glaucomatous optic atrophy are visible. Postoperative intraretinal hemorrhages can also be seen.

**Figure 5:**
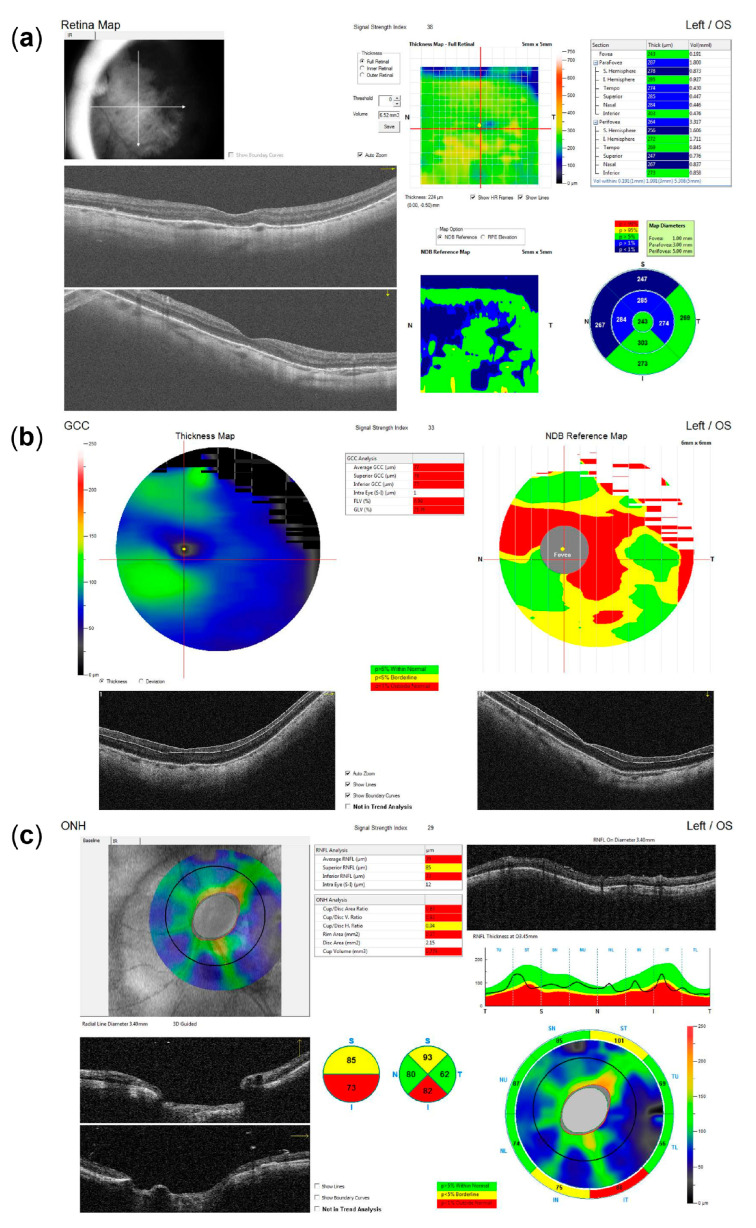
Optical coherence tomography (OCT) imaging following recovery from combined cataract surgery and vitrectomy, indicating glaucomatous optic neuropathy as the most likely primary cause of reduced visual acuity. (**a**) Macular OCT reveals no evidence of edema or other foveal pathology. (**b**) Ganglion cell complex (GCC) analysis demonstrates diffuse thinning, including the entire papillomacular bundle (note: segmentation artifacts that may underestimate GCC loss in the inferior portion of the papillomacular bundle may be observed in the horizontal B-scan). (**c**) The optic nerve head (ONH) OCT shows inferotemporal retinal nerve fiber layer (RNFL) thinning (note: reduced RNFL reflectivity and internal limiting membrane (ILM) thickening may be observed in the circular B-scan, contributing to segmentation artifacts that may underestimate the extent of overall RNFL loss).

**Figure 6:**
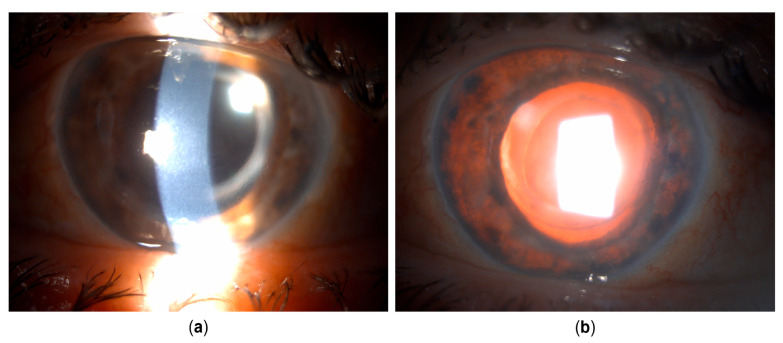
Clinical appearance several weeks after cataract surgery and pars plana vitrectomy. (**a**) Corneal edema developed. Pigmented corneal precipitates are no longer present. (**b**) Iris transillumination defects are now present circularly.

After three months of the documented absence of anterior chamber cells and complete resolution of keratic precipitates, with ongoing antiviral, corticosteroid, and IOP-lowering therapy, the patient underwent combined phacoemulsification cataract surgery with pars plana vitrectomy to remove the pigment obstructing the visual axis. Despite an unremarkable surgical course, the eye was hypotonic on postoperative day one, with multiple choroidal detachments and several intraretinal hemorrhages. While the hemorrhages persisted longer, the choroidal detachments and hypotony resolved within days (Figure 4), with BCVA improving only to 20/125. Optical coherence tomography (OCT) revealed no macular edema or overt foveal pathology (Figure 5). The thin extrafoveal hemorrhages (not visible in the central OCT scans), primarily localized to the outer plexiform layer with occasional extension into the subretinal space, may have indicated ischemia of the deep retinal capillary plexus. Nevertheless, as far as could be determined from the available scans, no definitive hyperreflective bands or thinning within the inner nuclear, outer plexiform, or outer nuclear layers were identified, and these layers appeared uniformly thick. A few pinpoint hyperreflective foci within the outer nuclear layer and outer photoreceptor segments, also extrafoveal, may have indicated focal ischemia at the level of the choriocapillaris. However, the outer nuclear layer, external limiting membrane, ellipsoid zone, and RPE/Bruch’s membrane complex appeared normal and continuous within the fovea. The most striking finding was the diffuse thinning of the ganglion cell complex, including the papillomacular region (Figure 5). The optic nerve head OCT demonstrated a glaucomatous pattern of retinal nerve fiber layer thinning, with inferotemporal involvement extending into the papillomacular bundle, likely related to a more oblique angle between the optic disc and the fovea (Figure 5). These findings supported glaucomatous optic neuropathy with a papillomacular bundle defect as the primary cause of the reduced BCVA, with possible additional functional impairment from the generalized hypoperfusion of the retinal and choroidal vascular beds. The IOP measurements continued fluctuating between 16 and 30 mmHg, despite ongoing IOP-lowering therapy, and as reliable IOP assessment was compromised due to the post-LASIK status of the eye, further clinical decisions were aided by digital tonometry and monitoring the status of the optic nerve head via fundoscopy and OCT. Within several weeks, however, signs of corneal decompensation with epithelial edema developed (Figure 6), reducing BCVA to counting figers (CF) at 1 meter. As no signs of inflammation were present, with antiviral and anti-inflammatory therapy still ongoing, and as recent specular microscopy had shown normal corneal endothelial morphology, this decompensation was considered likely secondary to elevated IOP. The patient subsequently underwent an Ahmed glaucoma valve implant. Postoperatively, IOP values were reduced to measurements between 6 and 13 mmHg, with only a partial resolution of corneal edema, and BCVA improved to 20/400. The patient was maintained on antiviral therapy (acyclovir 800 mg five times daily for two postoperative months, followed by a reduced dose of 400 mg twice daily for long-term prophylaxis), along with additional IOP-lowering treatment, with close monitoring for further changes in corneal edema and IOP.

## 3. Discussion

Herpetic uveitis can lead to significant pigment dispersion and iris transillumination defects, especially in cases with systemic immunosuppression, where bilateral presentation may also occur [3]. In such cases, the distinction between herpetic uveitis and acute iris transillumination syndrome can become problematic by means of clinical signs alone, and indicative medical history is instrumental. So far, as we know it, only a few acute iris transillumination-like cases have been described with confirmed HSV association [15,16]. This contrasts with the vast majority of BAIT/UAIT cases, which seem to have a distinct etiopathogenesis, primarily implicating the toxic effect of fluoroquinolones or other drugs on iris pigment epithelium [7]. And although viral etiology, including the herpesvirus infections, has also been suggested to play a role in BAIT/UAIT, conclusive evidence is lacking, with anterior chamber PCR for the herpesvirus family being negative in almost all published cases [4,5,6,12,17]. A conclusion could be drawn that cases with confirmed HSV association may be misinterpreted as falling within the scope of iris transillumination syndrome due to not being seen in the inflammatory phase, such as in the case described by Gonzalez Martinez et al. [15], or due to significant discrepancy between the amount of iris epithelial pigment dispersion and signs of inflammation, as in the case reported by Dastrup et al. [16]. This is reinforced by the reported history, although non-descriptive, of preceding acute iritis in the former case, and by the finding of keratic precipitates in the latter case. Both studies acknowledge herpetic etiology as uncommon for this syndrome. However, given the suggested role of viral infections in BAIT/UAIT, these studies imply the possible existence of a common pathway for virally mediated iris pigment loss. Our case also illustrates such a possibility through a well-documented shift from a clinical picture typical of herpetic uveitis to one suggestive of UAIT. However, while the apparent absence of inflammatory cells in the anterior chamber and vitreous during the latter phase could have suggested this alternative diagnosis, it is more likely that corticosteroid treatment masked these clinical findings, thereby blurring the distinction between the two entities.

In our case, a suggestive clinical presentation and a supportive medical history led to the diagnosis of herpetic anterior uveitis, which was further reinforced by PCR testing. However, while aqueous PCR testing is a highly specific and valuable diagnostic tool for identifying viral etiologies in anterior uveitis, its sensitivity can vary depending on factors such as the timing of sampling relative to symptom onset, viral load, and prior antiviral treatment [2], with reported detection rates ranging from 81% with real-time PCR to as low as 50–55% using conventional PCR, highlighting the need for clinical correlation when interpreting the results [18,19,20].

Regardless, the unabated progression of IPE atrophy and pigment dispersion during the phase with no visible inflammation suggests a distinct pathophysiological process either unrelated to general inflammatory activity, such as the direct viral cytopathic effect, or related to its inappropriate modulation. This opens up the possibility of the iatrogenic exacerbation of such processes. As previously mentioned, herpetic uveitis under systemic immunosuppression can manifest bilaterally and with more severe sequelae, as corticosteroid treatment without adequate antiviral coverage may reduce the host’s ability to control viral replication, potentially exacerbating cytopathic damage [3]. The delayed initiation of antiviral therapy in herpetic anterior uveitis can result from diagnostic challenges, errors, and under-recognition in both typical and atypical cases, leading to inappropriate corticosteroid monotherapy and increasing the risk of recurrences and long-term complications [3,21,22,23,24]. The lack of standardized guidelines further contributes to variability in treatment initiation, agent selection, administration route, dosage, and duration [23]. Although systematic studies quantifying undertreatment in real-world settings are lacking, the authors’ clinical impression is that these challenges remain relevant, particularly at the primary care and general ophthalmology levels. This underscores the need for heightened clinical suspicion and structured antiviral stewardship, especially given the potential for antiviral resistance in immunocompromised patients, which can range from 2.5% to over 30% depending on immunosuppression severity and underlying conditions [24].

It is noteworthy that manifestations of ocular herpetic disease such as retinal necrosis or iris atrophy are believed to result from a combination of direct viral effects on multiple cell types, including endothelial cells, and immune-mediated inflammation, leading to occlusive vasculitis [25,26]. Although our patient was treated only with topical and subconjunctival steroids, and was otherwise immunocompetent, these were at first used without appropriate antiviral treatment. This may have led to uncontrolled viral replication and delayed exacerbation mimicking UAIT due to cytopathic or ischemic damage and the necrosis of the IPE. While we did not obtain intraoperative iris samples for histological analysis in our study, in the study of Gonzalez Martinez et al., the histological examination of an enucleated eye demonstrated a pronounced absence of the IPE and iris dilator muscle, with extensive necrosis and focal vascular occlusion of the iris sphincter muscle, but no evidence of active vasculitis or nuclear viral cytopathic changes typical of herpesvirus infection [15]. Additionally, the inflammatory infiltrate in the uveal tract was extremely mild and predominantly lymphocytic. The authors noted possible limitations due to sampling, and the possible modification of histological findings due to topical steroids administered to the patient. It should be emphasized that their patient presented in the late, quiet phase, several months after the initial iritis episode (n.b. also treated with topical corticosteroids and without antiviral therapy), which likely influenced the histological findings.

HSV-1 infection can also cause mitochondrial DNA depletion and dysfunction through the action of the UL12.5 isoform, leading to increased oxidative stress and impaired mitochondrial energy production [27]. Fluoroquinolones, in turn, can form a chelate with essential cations such as magnesium and calcium, disrupt the electron transport chain, reduce mitochondrial membrane potential, and increase reactive oxygen species production within the mitochondria [28]. These mechanisms result in disrupted ATP synthesis, oxidative damage, and disturbances in cellular homeostasis, which may trigger apoptotic pathways and contribute to the pigment epithelium damage and acute pigment dispersion observed in both HSV-1 and fluoroquinolone-induced cases. Given this overlap, targeted antioxidant therapies may be considered as supportive strategies. Agents such as alpha-lipoic acid, coenzyme Q10, N-acetylcysteine, and vitamins C and E have been described to help stabilize mitochondrial function, lower oxidative stress, and support tissue recovery. However, caution is warranted, as the clinical benefit, timing, and dosing of antioxidants in these indications remain unproven, and excessive antioxidant use during viral illnesses could disrupt the physiological redox signaling or immune responses necessary for viral control [28]. Further research is needed to elucidate the role of these mitochondrial and oxidative stress mechanisms in the pathogenesis of both these clinical entities, and to clarify whether and how antioxidant support might be integrated into the comprehensive management of these complex cases.

## 4. Conclusions

Both our case and the previously mentioned cases highlight the unusual presentation of HSV-1 uveitis with acute iris transillumination-like findings. However, this observation only suggests a potential pathway for HSV-1-mediated IPE loss, which could be shared with other viruses, though this remains theoretical. Also, current clinical evidence for acute iris transillumination syndrome favors an alternative, toxic mechanism as the likely cause of IPE damage, distinguishing it from the mechanism postulated for HSV-1-related IPE loss. Therefore, it would be appropriate to use the descriptor “acute iris transillumination-like” in such cases, clearly differentiating between these entities. When clinical signs alone are insufficiently distinctive, aqueous tap and PCR testing for herpesviruses can provide additional diagnostic support. However, given the possibility of false-negative results, PCR testing should be viewed as confirmatory rather than exclusionary, and an indicative medical history should remain instrumental. More importantly, the potential for complications, such as those described here, arising from the use of corticosteroid treatment alone or from the delayed initiation of antiviral therapy, should be carefully considered. It may be prudent to apply a low threshold for the immediate introduction of antiviral therapy in any case of uveitis unattributed to other clear causes, particularly if clinical signs are consistent with possible herpetic etiology. Additionally, testing for antiviral resistance should be made readily available to allow for the timely adjustment of therapy in complicated or unresponsive cases.

## Data Availability

The authors declare that all data supporting the report are available upon request to the corresponding author.
